# Identification of genes associated with multiple cancers via integrative analysis

**DOI:** 10.1186/1471-2164-10-535

**Published:** 2009-11-17

**Authors:** Shuangge Ma, Jian Huang, Meena S Moran

**Affiliations:** 1 School of Public Health, Yale University, New Haven, CT 06520, USA; 2 Department of Statistics and Actuarial Science, University of Iowa, Iowa City, IA 52242, USA; 3 Department of Therapeutic Radiology, Yale University, New Haven, CT 06520, USA

## Abstract

**Background:**

Advancement in gene profiling techniques makes it possible to measure expressions of thousands of genes and identify genes associated with development and progression of cancer. The identified cancer-associated genes can be used for diagnosis, prognosis prediction, and treatment selection. Most existing cancer microarray studies have been focusing on the identification of genes associated with a specific type of cancer. Recent biomedical studies suggest that different cancers may share common susceptibility genes. A comprehensive description of the associations between genes and cancers requires identification of not only multiple genes associated with a specific type of cancer but also genes associated with multiple cancers.

**Results:**

In this article, we propose the Mc.TGD (Multi-cancer Threshold Gradient Descent), an integrative analysis approach capable of analyzing multiple microarray studies on different cancers. The Mc.TGD is the first regularized approach to conduct "two-dimensional" selection of genes with joint effects on cancer development. Simulation studies show that the Mc.TGD can more accurately identify genes associated with multiple cancers than meta analysis based on "one-dimensional" methods. As a byproduct, identification accuracy of genes associated with only one type of cancer may also be improved. We use the Mc.TGD to analyze seven microarray studies investigating development of seven different types of cancers. We identify one gene associated with six types of cancers and four genes associated with five types of cancers. In addition, we also identify 11, 9, 18, and 17 genes associated with 4 to 1 types of cancers, respectively. We evaluate prediction performance using a Leave-One-Out cross validation approach and find that only 4 (out of 570) subjects cannot be properly predicted.

**Conclusion:**

The Mc.TGD can identify a short list of genes associated with one or multiple types of cancers. The identified genes are considerably different from those identified using meta analysis or analysis of marginal effects.

## Background

Microarrays have been extensively used to profile tissues on a genome-wide scale. Genes identified from microarray studies can be used as cancer markers for diagnosis, prognosis prediction, and treatment selection. As an example, microarray gene signatures have been used in breast cancer and lymphoma clinical practices [1]. In this article, we focus on microarray studies where gene expressions are measured along with certain cancer clinical outcomes. The goal of such studies is to identify genes with important impacts on the clinical outcomes of interest, which may include risk of developing cancer, cancer status, cancer survival, and response to treatment [2].

Analysis of cancer microarray data is challenging first because of the high dimensionality of gene expressions. In addition, unlike simple Mendelian diseases, development and progression of cancer are affected by the *joint *effects of multiple genetic defects. This in turn demands modeling the joint effects of a large number of genes in a single statistical model and makes analysis of one gene at a time (i.e, marginal gene effects) suboptimal. Moreover, out of a large number of genes surveyed, only a subset are cancer-associated. To discriminate those cancer-associated genes from noises, various filter, wrapper, and embedded statistical methods have been developed [3].

In most existing studies, attentions have been focused on analysis of a single dataset and identification of genes associated with a single cancer clinical outcome. Consider a hypothetical study where we are interested in identifying genes associated with development of breast cancer. Assume that there are five genes of interest: genes A-E. The goal of most existing studies corresponds to the first column of Table 1, which is to distinguish between cancer-associated genes A and B from noisy genes C, D, and E. In this article, we refer to such a gene selection study as "one dimensional". That is, selection is only carried out on the genes.

**Table 1:** A hypothetical study.

	Cancer Development		
Gene	Breast	Ovarian	Lung
A	X	X	X
B	X	X	
C		X	X
D			X
E			

"X" indicates an association between the corresponding gene and cancer development.

All cancer cells share two essential characteristics: uncontrolled growth and local tissue invasion or metastasis. In addition, there is strong evidence that certain cancers share common susceptibility genes. Examples include the BRCA1 and BRCA2 tumor suppressor genes, whose mutations are associated with the inherited forms of both breast and ovarian cancers [4]. Over-expression of the HER-2 oncogene has been reported in 10-40% of primary breast and ovarian tumors and is strongly associated with a poor clinical prognosis [5]. Gene WWOX is a tumor suppressor gene mutated in both breast and prostate cancers [6]. Gene ADH is associated with development of lung cancer and head/neck cancer [7,8]. The wound response signature, which is a breast cancer prognostic gene signature, also has predictive power for prognosis of lung cancer and prostate cancer [9]. Simultaneously examining multiple cancers and searching for their common genomic basis will enable us to identify more essential features of cancer and lead to a better understanding of the subtle connections among different types of cancers [10].

When studying a single type of cancer, genes can be categorized simply as either cancer-associated or not. Selection only needs to be conducted at the gene dimension. When studying multiple cancers, the categorization becomes more complicated. Consider the hypothetical study presented in Table 1. Suppose that, in addition to breast cancer, we are also interested in ovarian and lung cancers. Among the five genes, gene A is associated with all three types of cancers. Genes B and C are associated with two types of cancers. Gene D is associated with only one type of cancer, and gene E is not associated with any of the three cancers. Examination of Table 1 suggests that development of breast and ovarian cancers may share a common genomic mechanism, which likely involves the protein encoded by gene B. However, such a mechanism may have no effect on development of lung cancer. When multiple genes and multiple cancers are considered, selection needs to be carried out at two dimensions: (a) the gene dimension. For each type of cancer, genes associated with its development need to be identified. For example, for ovarian cancer, this dimension of selection amounts to differentiating genes A-C from genes D and E; and (b) the cancer dimension. For each gene, we are interested in identifying cancers it is associated with. For example, for gene B, this dimension of selection amounts to differentiating breast and ovarian cancers from lung cancer. Of note, although there are studies investigating multiple genes and multiple cancers, none of them formally considers this as a two-dimensional selection problem.

Studies conducted to identify genes associated with multiple cancers include [11], where 218 tumor samples spanning 14 common tumor types and 90 normal tissue samples were collected and analyzed to identify a gene signature that is differentially expressed in metastatic tumors of diverse origins relative to primary cancers. A "support vector machine + recursive feature elimination" approach is proposed. Such an approach is limited to categorical clinical outcomes. We note that the data structures and scientific questions of interest in [11] and their counterparts in this article are significantly different. More specifically, [11] has one multiclass classification problem, whereas we have multiple binary classification problems. Rhodes et al. [10] examined 21 cancer microarray datasets spanning 12 distinct cancer types and identified a set of 67 genes that are universally activated in most cancer types relative to normal tissues. The approach proposed in [10] can only study the marginal effects of genes, whereas cancer development is associated with the joint effects of multiple genetic defects. Segal et al. [12] pooled 1975 human DNA microarrays spanning 22 tumor types and characterized gene expression profiles in tumors as a combination of activated and deactivated modules. An approach similar to the Fisher's meta analysis approach is proposed, which can study the marginal effects of genes only. Chan and Mousavi [13] proposed a stochastic Bayesian approach to identify susceptibility genes shared by development of breast and ovarian cancers. The SHEBA approach demands selection of closely related cancers. Considering our limited knowledge of mechanisms beneath cancer development, potential applications of this approach can be limited. Yang et al. [14] analyzed 4 cancer prognosis studies involving breast cancer, leukemia, and mesothelioma and identified 42 genes that show consistent up- or down-regulation in patients with poor disease outcomes. An extension of the approach in [10] is considered, which can only study the marginal effects of genes. Xu et al. [15] collected 26 cancer datasets across 21 major human cancer types and identified a common cancer signature consisting of 46 genes. The proposed TSPG approach is limited to categorical clinical outcomes and hard to be extended. Choi et al. [16] analyzed 10 gene expression datasets from cancers of 13 different tissues and constructed two distinct coexpression networks: a tumor network and a normal network. This study focuses on analyzing the pair-wise interactions between genes. *Lê *Cao et al. [17] analyzed the NCI60 datasets, where the transcriptome of 60 cancer cell lines was investigated. The sparse partial least squares (sPLS) method was used, which cannot be easily extended to other data setup/models.

Existing methods for analyzing multiple cancer microarray datasets may have one or more of the following drawbacks. First, attention has been focused on analyzing one gene at a time (i.e, the *marginal *effects of genes). Examples include [10,12,14,16] and others. Since development and progression of cancer is caused by the joint effects of multiple genes, analyzing individual genes separately does not make full use of information in data. In this study, we include all genes in a single statistical model and account for their joint effects. Second, the focus has been on identification of genes associated with *all *cancers being investigated. Such a strategy demands preselection of cancers having a significantly overlapped genomic basis. For example, in [13], only breast cancer and ovarian cancer - which are known to share a common genomic basis - are investigated. This strategy may have significant limitations given the great heterogeneity among different cancers and our limited knowledge of cancer genomics. In this study, we release this constraint, and allow the data to reveal which cancers a particular gene may be associated with. Third, multiple datasets are usually analyzed separately. Then, summary statistics (for example p-values) from analysis of each individual dataset are combined using meta analysis methods to search for overlaps of findings. Such an approach can be inefficient since microarray studies have small sample sizes, and analyzing each individual dataset separately may have insufficient power and may lead to high false positive and false negative errors. Fourth, inefficient feature selection methods are employed. For example, in [15], the number of cancer-associated genes needs to be predetermined, and the heuristic exhaustive search approach in [13] can accommodate only a small number of genes.

In this article, we propose a new statistical approach - Mc.TGD (Multi-cancer Threshold Gradient Directed) - for investigation of associations between multiple genes and multiple cancers. The Mc.TGD is an integrative analysis approach in which *raw data *from multiple studies are pooled and analyzed. It differs significantly from meta analysis methods, which analyze each dataset separately and pool *summary statistics*. Unlike existing approaches, the Mc.TGD can model the joint effects of multiple genes, does not make assumptions on the genomic basis of cancers, uses effective gene selection techniques, and is broadly applicable. In this article, we analyze studies investigating the risk of developing cancer, which have binary outcomes. The Mc.TGD can also be used to analyze cancer microarray studies with survival, quantitative, and categorical outcomes.

## Results

### Data collection

As shown in Table 2, we collect data from seven studies conducted by different research groups who investigated cancers of different tissues and used different profiling platforms.

**Table 2:** Description of datasets.

Tissue	Reference	Platform	Normal	Tumor
Breast	Sorlie et al. (2001) [27]	cDNA	13	13
Kidney	Boer et al. (2001) [38]	membrane	81	81
Liver	Chen et al. (2002) [39]	cDNA	76	76
Lung	Bhattacharjee et al. (2001) [40]	U95A	17	17
Pancreas	Iacobuzio-Donahue et al. (2003) [41]	cDNA	14	22
Prostate	Singh et al. (2002) [42]	U95A	50	52
Stomach	Chen et al. (2003) [43]	cDNA	29	29

The normalized datasets have been downloaded from the Stanford Microarray Database [18] and NCBI [19]. These seven datasets have also been investigated in [16], where three more datasets are analyzed. Including these three additional datasets leads the number of genes measured in all studies to decrease from 2207 to 371. To keep a reasonable number of genes, only the seven studies described in Table 2 are analyzed. Of note, although this study and [16] analyze similar datasets, the two studies differ significantly in that ours analyzes multiple genes at a time and seeks to identify those with important joint effects. In contrast, [16] analyzes one gene at a time. Thus, the two studies are not directly comparable. Rather, they investigate different aspects of genes and complement each other.

The following data processing is conducted for each dataset separately. Negative values of Affymetrix measurements are considered as missing. Genes with more than 70 missing values are filtered out. All of the expression values are log 2 transformed. Each clone is mapped to a UniGene accession based on UniGene build # 162. For multiple clones matched to the same UniGene accession, the one with the least missing values is chosen. Missing measurements are imputed using the means of gene expressions across samples. For each dataset, each gene expression is normalized to zero mean and unit variance. A consensus set of 2207 genes are identified.

For the breast, liver, lung and stomach cancer datasets, the tumor sample sizes were much larger than the normal sample sizes. We conduct the same selection as in [16], which leads to an equal number of tumor and normal samples.

### Gene identification

We analyze the seven datasets using the Mc.TGD. With 5-fold cross validation, (*τ*_1_, *τ*_2_, *k*) = (1.0, 0.85, 1311) are selected as the optimal tuning parameters. Gene identification results, including UniGene identifiers, gene names, and estimated coefficients, are shown in the Additional File 1. With the Mc.TGD approach, we conclude an association between a gene and cancer, if and only if a nonzero estimated regression coefficient is observed. A total of 60 genes are identified to be associated with one or more types of cancers.

Gene MT1F (UniGene Hs.438737) is found to be associated with six types of cancers (all except breast cancer). The MT1F gene belongs to the metallothionein (MT) family, which encodes a family of cysteine-rich, low molecular weight proteins. Published studies on MT1F have shown an association between this gene and a protective effect against metal toxicity, involvement in the physiologic regulation of metals such as zinc and copper, and a role in protection against oxidative stress. Since MTs play an important role in transcription factor regulation, problems with MT function or expression may lead to cellular changes that ultimately result in transformation to malignant cells. Studies have found increased expressions of MTs in cancers of the breast, colon, kidney, liver, lung, nasopharynx, ovary, prostate, mouth, salivary gland, testes, thyroid, and urinary bladder. Early studies have also found lower levels of MT expressions in hepatocellular carcinoma and liver adenocarcinoma. Moreover, there is evidence to suggest that higher levels of MT expressions may lead to resistance to chemotherapeutic drugs. We refer to [20-26] for studies that have identified MT1F as a marker for various cancers. Although MT1F has been previously identified as a marker for breast cancer, our study is unable to identify its association with breast cancer using the data from [27]. There are multiple possible reasons, including the small sample size, quality of data, and possible limitations of the Mc.TGD.

Four genes are found to be associated with five types of cancers. Gene Hs.15154 is sushi-repeat-containing protein, X-linked (SRPX). Its role in cancer development has not been well investigated. Gene Hs.1560 is DNA cross-link repair 1A (PSO2 homolog, S. cerevisiae) with official symbol DCLRE1A. DNA interstrand cross-links prevent strand separation, thereby physically blocking transcription, replication, and segregation of DNA. DCLRE1A is one of several evolutionarily conserved genes involved in repair of interstrand cross-links [28]. It regulates BRCA1, the obnoxious breast cancer susceptibility gene [29]. In mice models, it has been shown that DCLRE1A co-regulates with IGF-I. Suppression of IGF-I is associated with a low incidence of kidney disease [30]. In addition, a significant association between DCLRE1A and the development of lung cancer has been observed [31]. Gene Hs.418083 (official symbol RBP4) is retinol binding protein 4. This protein belongs to the lipocalin family and is the specific carrier for retinol in blood. It delivers retinol from the liver stores to the peripheral tissues. RBP4 level can be used as an index of cardiovascular disease risk in subclinical hypothyroidism. Retinol binding protein 4 may contribute to the pathogenesis of nonalcoholic fatty liver disease in type 2 diabetics. Gene Hs.435330 (official symbol KIAA0372) has not been well investigated.

In addition, 11 genes are found to be associated with four types of cancers. 9, 18, and 17 genes are found to be associated with three, two, and one types of cancers, respectively. Many of these genes have been previously identified as cancer markers in independent studies.

### Evaluation

We evaluate prediction performance of the Mc.TGD identified genes. Since we do not have independent studies with comparable designs, we use the Leave-One-Out (LOO) cross validation evaluation [32].

The LOO approach consists of the following steps: (a) Subject *j *(= 1 ... *n*_*m*_) is first removed from study *m *(= 1 ... *M*). Here *M *denotes the total number of studies and *n*_*m *_is the number of subjects in study *m*; (b) The Mc.TGD estimated regression coefficient  with the reduced data is computed. To have a fair evaluation, we need to select a new set of tuning parameters for the reduced data; (c) For the removed subject, compute the risk score as , where *z*_*m*, *j *_is the vector of gene expressions for subject *j *in study *m*; (d) Repeat Steps (a)-(c) over all studies and all subjects; (e) For each subject, a predictive probability can be computed using the logistic model; (f) Dichotomize the predictive probabilities at 0.5 and make predictions. Prediction performance can then be evaluated by comparing predictive and observed cancer status.

With the LOO approach, only four subjects in the lung cancer study are not properly classified, which leads to an overall error rate of 0.7%. The LOO evaluation is cross validation based. Since a new set of tuning parameters and estimates are computed with each reduced data, the LOO approach is expected to be relatively fair.

### Meta analysis

For comparison, we consider the following meta analysis approach. We first analyze each study separately using the TGDR approach [33,34] and then search for genes that are identified in multiple studies. This meta analysis approach uses the voting method to combine analysis results from multiple studies. We are aware that the TGDR can be replaced by other regularization approaches. However, multiple studies have shown that it performs comparably to other single-dataset approaches [33-35]. Furthermore, unlike other regularization approaches, the TGDR has a thresholding framework similar to that of the Mc.TGD and is therefore chosen for comparison.

With this approach, a total of 181 genes are identified to be cancer-associated. However, only four genes are found to be associated with two cancers. All the other genes are found to be associated with only one type of cancer. Compared to this approach, the Mc.TGD is able to take information from multiple studies into consideration in gene selection and thus is more effective in identifying genes that are associated with multiple cancers.

### Analysis of marginal associations

With the Mc.TGD, we describe effects of multiple genes using a single statistical model and thus are able to account for their joint effects. To provide a more comprehensive description of identified genes, we also conduct the following analysis of marginal associations: (a) For each gene in each study, we use the Wilcoxon rank-sum test to compare gene expressions of cancer patients with those of normal patients; (b) We then rank genes using their p-values. The gene with rank 1 has the smallest p-value. This approach shares similar spirits as those for detection of differentially expressed genes in [10,12,14,16].

genes identified with the Mc.TGD, we show their marginal ranks in the Additional File 1. We found that genes identified as jointly associated with cancers not necessarily have high marginal ranks. For example, gene MT1F is identified to be associated with six types of cancers. However, its marginal ranks are only 532, 71, 54, 336, 25, and 28, respectively. This finding confirms the necessity of identifying genes with joint effects beyond analysis of marginal effects.

## Discussion

When implementing the Mc.TGD, we focus on genes measured in all studies. As an alternative, when different studies have overlapped but different sets of genes, we can impute gene expressions not measured as zero, and then apply the Mc.TGD. An important objective of the Mc.TGD is to identify genes associated with multiple or all cancers investigated. The proposed analysis can be increasingly unreliable as the number of overlapped genes decreases. Focusing on genes measured in all studies may pose a limitation to the proposed analysis. However, in the very near future, when pangenomic arrays become routine, this limitation may no longer be an issue.

The Mc.TGD analyzes multiple cancer microarray datasets. The final output may be unreliable if one or more datasets have low qualities. In practical implementation, careful inspection of each individual dataset is imperative.

In this study, we evaluate the identified genes in two different ways. First, for those identified to be associated with six and five types of cancers, we manually search published literature for existing evidences of them being associated with cancer. Second, we use the LOO approach and evaluate the overall prediction performance of the Mc.TGD and identified genes. As one reviewer pointed out, our evaluation is still far from complete. To fully evaluate the sixty identified genes, independent biomedical studies may be needed, and that is beyond the scope of this article.

In our data analysis, we focus on studies that investigate the risk of developing cancer. Such studies have binary outcomes and can be naturally described using logistic models. As can be seen from the Methods section, the Mc.TGD is also applicable to other cancer clinical outcomes. More specifically, with continuous clinical outcomes, we can use linear regression models. With multiclass categorical outcomes, we can use generalized liner models. With censored survival outcomes, we can use the Cox proportional hazards model. Once statistical models are specified, likelihood functions can be constructed, and the Mc.TGD can be employed.

## Conclusion

A large number of cancer microarray studies have been conducted to search for genes associated with development and progression of various types of cancers. Compared with genes associated with a single type of cancer, genes associated with multiple cancers can represent the more essential genomic features of cancer. In this article, we propose Mc.TGD, an integrative analysis approach that can pool and analyze raw data from multiple studies on different types of cancers. Although there are other studies investigating associations between genes and multiple cancers, the Mc.TGD is the first embedded approach to conduct "two-dimensional" selection and account for the joint effects of genes in such selection. Compared with existing approaches, the Mc.TGD can provide a much more comprehensive description of gene effects on cancer.

Seven cancer microarray studies are analyzed. A total of sixty genes are identified. For genes MT1F, DCLRE1A, RBP4, and many others, the identified associations are consistent with findings in the literature. For other genes, such as SRPX and KIAA0372, more biomedical studies are needed to fully understand their roles in cancer. The LOO evaluation suggests satisfactory prediction performance, which provides support for the identified associations. Ideally, prediction evaluation using completely independent data is needed to confirm the findings. However, this is beyond the scope of this article.

## Methods

Our proposed approach for detecting genes associated with multiple cancers consists of the following steps: (a) With each dataset, model the joint effects of all genes on cancer clinical outcome using a regression model; (b) Since multiple datasets on multiple cancers are being investigated, define the overall objective function, which measures the overall association between genes and cancer clinical outcomes; and (c) Apply the Mc.TGD, which is an iterative, two-dimensional selection approach. At each iteration, for each gene, the Mc.TGD evaluates its overall effect to determine if it is associated with any cancer, as well as individual effects on each cancer to determine which cancer type(s) it is associated with.

### Data and model

Consider *M *> 1 studies that measure clinical outcomes of possibly different cancers. For simplicity of notations, suppose that the same set of *d *genes are measured in all *M *studies. For the datasets presented in Table 2, *M *= 7 and *d *= 2207. Let *Y*_1_,..., *Y*_*M *_denote the cancer clinical outcomes, and *Z*_1_,..., *Z*_*M *_denote the *d *gene expressions in study 1 ... *M*. In this article, we study the risk of developing cancer, where the outcome is the binary cancer status. In study *m*, we use *Y*_*m *_= 1 or 0 to denote the presence or absence of cancer.

For each individual dataset, we use the logistic regression model to describe the effects of genes on the binary cancer outcome. For study *m*, *logit*(*P*(*Y*_*m *_= 1|*Z*_*m*_)) = *α*_*m *_+ *β*_*m*_, where *α*_*m *_is the unknown intercept,  is the transpose of *Z*_*m*_, and *β*_*m *_is the length *d *regression coefficient. Based on a sample of *n*_*m *_iid observations, the log-likelihood function is . In what follows, the log-likelihood will be used as the function to be maximized with the Mc.TGD and will be referred to as the objective function.

### Regularized gene selection

The Mc.TGD is an embedded approach, which embeds selection in model fitting [3]. Selection amounts to properly estimating the regression coefficients in logistic models.

Let *β *= (*β*_1_,..., *β*_*M*_) be the *d *× *M *matrix of regression coefficients. Denote *R*(*β*) = *R*_1_(*β*_1_) + ... + *R*_*M*_(*β*_*M*_) as the overall objective function. Denote Δ*ν *as the small positive increment in the gradient searching. In our numerical implementation, we set Δ*ν *= 10^-3^. With fixed thresholds 0 ≤ *τ*_1_, *τ*_2 _≤ 1:

1. Initialize *β *= 0 component-wise.

2. Compute the *d *× *M *gradient matrix , where its (*i*, *j*)^*th *^element is . Here *β*_*i*, *j *_is the *i*^*th *^element of *β*_*j*_.

3. Compute the length *d *cross-gene gradient *G*, where its *i*^*th *^component is . Compute the length *d *cross-gene thresholding vector *T*_*G*_, where its *i*^*th *^component is *T*_*G*, *i *_= *I*(*G*_*i *_≥ *τ*_1 _× *max*_*l*_*G*_*l*_).

4. For gene *i *= 1, ..., *d*, compute the length *M *cross-cancer thresholding vector , where its *m*^*th *^component is  = *I*(|*g*_*i*, *m*_| ≥ *τ*_2 _× *max*_*l*_|*g*_*i*, *l*_|).

5. Update *β*_*i*, *j *_with *β*_*i*, *j *_+ Δ*ν *× *g*_*i*, *j *_× *T*_*G*, *i *_× .

6. Steps 2 to 5 are repeated *k *times, where *k *is determined with cross validation.

The Mc.TGD uses *thresholding *to remove noisy genes and carry out gene selection. In Step 1, the Mc.TGD starts with no genes identified as cancer-associated. In Step 2, the gradients are computed. For each study, the gradients measure the strengths of associations between the genes and cancer clinical outcomes. Genes with stronger associations will have larger gradients. To make different genes comparable, their expressions have been normalized to have unit variances. In Step 3, the cross-gene gradients and the corresponding thresholding vector are computed. In this step, the *overall *association of a gene with all the cancer outcomes is measured. By introducing the threshold, we compare one gene with the rest of the genes. Genes with more combined strengths of associations with all cancers will have the corresponding components of *T *equal to one. In Step 4, for each gene, its gradients - strengths of associations with individual cancer clinical outcomes - are computed. By introducing the cross-cancer thresholding vector, we can identify those cancers this gene is associated with. In Step 5, the two thresholds are combined, allowing the determination of not only whether a particular gene is associated with any type of cancer at all but also which specific cancer type(s) this particular gene is associated with. The estimate is updated if and only if an association is observed. The iterations continue until terminated by cross validation.

To further illustrate, we consider the hypothetical study presented in Table 1. Genes A-D are associated with one or more types of cancers, whereas gene E is not. The cross-gene gradients for genes A-D will be larger than that for gene E. Thus, with the thresholding in Step 3, we are able to discriminate gene E from others. Furthermore, consider gene B as an example. Gene B is associated with development of breast and ovarian cancer but not lung cancer. The gradient for gene B in the lung cancer study will be considerably smaller than those in the breast and ovarian cancer studies. Thus, with the thresholding in Step 4, we can identify gene B as a susceptibility gene for breast and ovarian cancers but not for lung cancer. By combining Steps 3 and 4, we are able to construct a complete description of gene effects as shown in Table 1.

#### Remarks: connections with existing methods

The Mc.TGD belongs to the family of embedded selection methods [3]. It shares the "computing (gradients), searching (for covariates that can increase value of the objective function), and updating (estimates of selected covariates)" framework with the gradient boosting and individual-dataset TGDR approaches [33,36]. Like many other regularization methods, the Mc.TGD determines the existence of associations by examining the estimated regression coefficients. A nonzero estimated regression coefficient indicates existence of an association, which is equivalent to a thresholding approach with zero as the threshold.

Among the many available selection methods, the TGDR [33] and the MTGDR [37] have a statistical framework closest to that of the Mc.TGD. More specifically, all three methods are iterative and use the thresholding technique for selection. The Mc.TGD significantly advances from the TGDR by being able to analyze multiple datasets, whereas the TGDR is a single-dataset method. Our numerical studies suggest that, analyzing individual datasets separately using the TGDR and then combing the results using meta analysis is suboptimal. Both the MTGDR and Mc.TGD can analyze multiple datasets. However, only the Mc.TGD can carry out two-dimensional selection. Compared with the MTGDR, the Mc.TGD has the extra cross-cancer thresholding (Step 4), which can identify cancer(s) a gene is associated with. Consider for example gene B in Table 2. With the Mc.TGD, the estimated coefficient in the lung cancer study will be exactly zero. Thus, we are able to conclude that gene B is associated with breast and ovarian cancers but not lung cancer. However, if the MTGDR is applied, since it does not have the cancer-dimension selection, the estimated coefficients in all three studies will be nonzero. The Mc.TGD, MTGDR, and many other regularization methods (for example penalization methods) use zero as cutoff to determine existence of associations. So even though the estimated coefficient for gene B in the lung cancer study may be very small, we do not have the technique to determine that the observed small coefficient does not represent a real association. This is the main reason why the Mc.TGD is needed beyond the MTGDR. In summary, we propose using the MTGDR when multiple datasets are on the same cancer and have the same set of susceptibility genes; In contrast, the Mc.TGD should be adopted when multiple datasets are on different cancers with different sets of susceptibility genes.

#### Remarks: possible extensions

In this study, we use the Mc.TGD to analyze seven datasets on seven different types of cancers. In other studies, it is possible out of the multiple datasets, two or more have similar designs (e.g., same cancer clinical outcome, same set of genes, same platforms, comparable cohorts). Then it may be reasonable to assume that the sets of identified genes are identical across those studies. In that case, for each gene, we can add an extra constraint to make components of the cross-cancer thresholding vector corresponding to those studies equal.

### Tuning parameter selection

Consider the association table, which is a table similar to Table 1 and the Additional File 1 and shows all the associations between cancers and genes. When the table is sparse, few genes are identified as cancer-associated; and for a specific gene, associations with few cancers are identified. When the table is dense, more associations are identified.

The Mc.TGD approach involves three tuning parameters: *k, τ*_1 _and *τ*_2_, which jointly determine sparsity of the association table. More specifically, with fixed (*τ*_1_, *τ*_2_), the table is sparse with small *k *and gets denser as *k *increases. When (*τ*_1_, *τ*_2_) are small, the table can be dense even with small *k*. In contrast, when (*τ*_1_, *τ*_2_) are close to one, the table is sparse with small to moderate *k*, but eventually becomes dense as *k *increases.

We select tuning parameters using V-fold cross validation [36]. To facilitate computing, we search over the discrete grid of *τ*_1_, *τ*_2 _= 1, 0.95, 0.9 ... 0.05, 0. We first randomly partition each dataset into *V *nonoverlapping subsets with equal sizes. Denote *β*^-*v *^as the Mc.TGD estimate of *β *based on data without the *v*^*th *^subset of each dataset. The CV objective function is defined as *CV *(*k, τ*_1_, *τ*_2_) = ∑_*v *_*R*^*v*^(*β*^-*v*^), where *R*^*v *^is the overall objective function evaluated on the *v*^*th *^subsets. Optimal tuning parameters are defined as (*k, τ*_1_, *τ*_2_) that maximize the CV objective function.

#### Remarks: Why is cross validation needed

Although each Mc.TGD iteration increases value of the overall objective function, the iteration needs to be terminated within a finite number of steps. Otherwise, with the number of genes larger than the sample size, there is a possibility of overfitting, where value of the overall objective function goes to infinity. In addition, a larger value of the overall objective function does not indicate a better prediction performance of identified genes. Thus, we use cross validation and choose the tuning parameters (particularly finite *k*) that maximizes the cross-validated prediction.

#### Remarks: an ad hoc alternative

In some cases, researchers may have certain prior information on sparsity of the association table. For example, researchers may have an estimate of the number of cancer-associated genes or only want to investigate a fixed number of genes. Then, instead of using cross validation, researchers may directly apply the Mc.TGD and terminate the iteration once a certain number of genes are identified.

### Parameter paths

To provide a graphic description of the Mc.TGD, we examine its parameter paths (estimates as a function of the number of iterations). We simulate under Scenario 3 presented in Table 3. Two datasets are generated, both with binary outcomes. In each dataset, there are 500 genes and 50 subjects with about an equal number of subjects having *Y *= 1 and 0. Genes 1 to 10 are associated with the first type of cancer, and genes 6 to 15 are associated with the second type of cancer. The two types of cancers share 5 common susceptibility genes. The rest are noisy genes.

**Table 3:** Simulation study: mean counts based on 200 replicates.

Scenario	# gene	**coef**.	Approach	Pos. 1	Pos. 2	TP 1	TP 2	Overlap
1	20	0.25	Mc.TGD	13	12	10	10	5
			TGDR	15	16	10	10	5
2	20	0.35	Mc.TGD	12	12	10	10	5
			TGDR	14	14	10	10	5
3	500	0.25	Mc.TGD	28	27	9	10	5
			TGDR	35	35	10	9	4
4	500	0.35	Mc.TGD	24	25	10	9	5
			TGDR	34	34	10	9	5
5	1000	0.25	Mc.TGD	31	31	9	9	5
			TGDR	39	38	9	9	4
6	1000	0.35	Mc.TGD	30	30	10	9	5
			TGDR	37	38	10	9	5

Pos. 1 (2): number of genes identified to be associated with cancer 1 (2); TP 1 (2): number of true positives for cancer 1 (2); Overlap: number of genes identified to be associated with both cancers.

**Figure 1:**
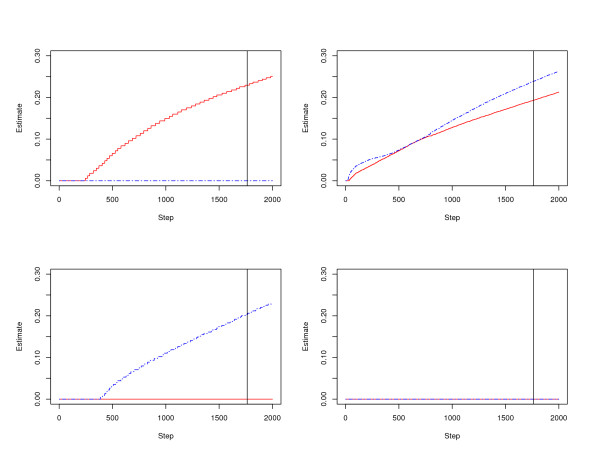
**Parameter paths of the Mc.TGD estimates**.

The simulated datasets are analyzed with the Mc.TGD. With five-fold cross validation, the optimal tuning (*τ*_1_, *τ*_2_, *k*) = (0.9, 0.9, 1763). In Figure 1, we show the parameter paths as a function of *k *for (*τ*_1_, *τ*_2_) = (0.9, 0.9), with the vertical lines corresponding to *k *= 1763. For the purpose of clarity, only parameter paths for genes #1, 6, 11, and 21 are presented.

As can be seen from Figure 1, parameter paths for different genes are significantly different. In the upper-right panel, we show the parameter paths for gene # 6, which is associated with both types of cancers. We can see that the estimated coefficients are nonzero for even very small *k*. In the upper-left and lower-left panels, we show the parameter paths for genes # 1 and 11, which are associated with only one type of cancer. We can see that the estimated coefficients are nonzero in only one study. In the lower-right panel, for gene # 21, which is not associated with any cancer, the estimated coefficients are zero. Since zero estimates indicate no association, Figure 1 suggests that we are able to correctly determine associations between multiple genes and multiple cancers by investigating properties of the Mc.TGD estimates or their parameter paths.

### Simulation study

Simulations are conducted to evaluate performance of the Mc.TGD. We assume that there are two studies on two different types of cancers. The benefit of simulating two studies is that the definition of associations between genes and cancers is lucid. As shown in Table 3, we consider the following simulation settings (a) number of genes: 20, 500 and 1000; (b) sample size: we set sample size equal to 50 in each study; and (c) regression coefficients. For genes associated with the outcomes, we set their regression coefficients equal to 0.25 or 0.35, which correspond to two different levels of signals. In addition, we set genes 1-10 to be associated with the first type of cancer, genes 6-15 to be associated with the second type of cancer, and the rest to be noisy genes. Two types of cancers share 5 common susceptibility genes. We generate gene expressions to be multivariate normally distributed and marginally with zero mean and unit variance. Expressions of genes *i *and *j *have correlation coefficient 0.4^|*i*-*j*|^. We generate the probability of cancer presence from the logistic regression model and then the cancer status from a binomial distribution. Under the present simulation settings, there are about equal number of subjects with *Y *= 1 and *Y *= 0. We simulate 200 replicates and show the summary statistics in Table 3. For comparison, we also consider the TGDR-based meta analysis described in the Results section. This approach is referred to as "TGDR" in Table 3.

With simulated data, we investigate how many genes are identified to be associated with one or both types of cancers. We can see from Table 3 that (a) under all simulated settings, the Mc.TGD is capable of identifying all genes associated with both types of cancers; (b) for each type of cancer, the Mc.TGD is capable of identifying a small number of genes and the majority or all of cancer-associated genes; (c) performance of the Mc.TGD improves as the number of genes decreases or as the signal (regression coefficients) increases; and (d) compared to the TGDR, the Mc.TGD has a lower false positive rate.

## Authors' contributions

All authors were involved in the study design and writing. SM and JH were involved in data analysis. All authors read and approved the final manuscript.

## Supplementary Material

Additional file 1 **Genes identified using the Mc.TGD**. The additional file contains information on all genes identified using the Mc.TGD: UniGene, gene names, and estimated regression coefficients. Click here for file

## References

[B1] Rhodes D Chinnaiyan AM Bioinformatics strategies for translating genome-wide expression analyses into clinically useful cancer markers Annals of the New York Academy of Sciences 2004 1020 32 40 10.1196/annals.1310.005 15208181

[B2] Knudsen S Cancer Diagnostics with DNA Microarrays 2006 Liss: Wiley

[B3] Ma S Huang J Penalized feature selection and classification in bioinformatics Briefings in Bioinformatics 2008 9 392 403 10.1093/bib/bbn027 18562478 PMC2733190

[B4] Petrucelli N Daly MB Culver JOB Feldman GL BRCA1 and BRCA2 hereditary breast/ovarian cancer GeneReviews 2007 Http://www.ncbi.nlm.nih.gov/bookshelf/br.fcgi?book=gene&part=brca1

[B5] Puputti M Sihto H Isola J Butzow R Joensuu H Nupponen NN Allelic imbalance of HER2 variant in sporadic breast and ovarian cancer Cancer Genetics and Cytogenetics 2006 167 32 38 10.1016/j.cancergencyto.2004.09.023 16682283

[B6] Qin H Iliopoulos D Semba S Fabbri M Druck T Volinia S Croce CM Morrison CD Klein RD Huebner K A role of the WWOX gene in prostate cancer Cancer Research 2006 66 6477 6481 10.1158/0008-5472.CAN-06-0956 16818616

[B7] Beckles MA Spiro SG Colice GL Rudd RM Initial evaluation of the patient with lung cancer Chest 2003 123 97S 104S 10.1378/chest.123.1_suppl.97S 12527569

[B8] Wang D Ritchie JM Smith EM Zhang Z Turek LP Haugen TH Alcohol dehydrogenase 3 and risk of squamous cell carcinomas of the head and neck Cancer Epidemiology, Biomarkers & Prevention 2005 14 626 632 10.1158/1055-9965.EPI-04-0343 15767341

[B9] Cheang M Rijn M van de Nielsen TO Gene expression profiling of breast cancer Annual Review of Pathology: Mechanisms of Disease 2008 3 67 97 10.1146/annurev.pathmechdis.3.121806.151505 18039137

[B10] Rhodes DR Yu J Shanker K Deshpande N Varambally R Ghosh D Barrette T Pandey A Chinnaiyan AM Large-scale meta-analysis of cancer microarray data identifies common transcriptional profiles of neoplastic transformation and progression PNAS 2004 101 9309 9314 10.1073/pnas.0401994101 15184677 PMC438973

[B11] Ramaswamy S Tamayo P Rifkin R Mukherjee S Yeang CH Angelo M Ladd C Reich M Latulippe E Mesirov JP Poggio T Gerald W Loda M Lander ES Golub TR Multiclass cancer diagnosis using tumor gene expression signatures 2001 98 15149 15154 11742071 10.1073/pnas.211566398 PMC64998

[B12] Segal E Friedman N Koller D A module map showing conditional activity of expression modules in cancer Nature Genetics 2004 36 1090 1098 10.1038/ng1434 15448693 PMC2271138

[B13] Chan C Mousavi P Discovery of gene expression patterns across multiple cancer types IEEE 5th Symposium on Bioinformatics and Bioengineering 2005 121 128 full_text

[B14] Yang X Bentink S Spang R Detecting common gene expression patterns in multiple cancer outcome entities Biomedical Microdevices 2005 7 247 251 10.1007/s10544-005-3032-7 16133813

[B15] Xu L Geman D Winslow RL Large-scale integration of cancer microarray data identifies a robust common cancer signature BMC Bioinformatics 2007 8 275 10.1186/1471-2105-8-275 17663766 PMC1950528

[B16] Choi JK Yu U Yoo OJ Kim S Differential coexpression analysis using microarray data and its application to human cancer Bioinformatics 2005 21 4348 4355 10.1093/bioinformatics/bti722 16234317

[B17] Lê Cao KA Martin PGP Robert-Graniê C Besse P Sparse canonical methods for biological data integration: application to a cross-platform study BMC Bioinformatics 2009 10 34 10.1186/1471-2105-10-34 19171069 PMC2640358

[B18] Stanford Microarray Database http://smd.stanford.edu/

[B19] National Center for Biotechnology Information http://www.ncbi.nlm.nih.gov/

[B20] Jin R Chow VT Tan PH Dheen ST Duan W Bay BH Metallothionein 2A expression is associated with cell proliferation in breast cancer Carcinogenesis 2002 23 81 86 10.1093/carcin/23.1.81 11756227

[B21] Lu D Chen Y Zhang X Cao X Jiang H Yao L The relationship between Metallothionein-1F (MT1F0 gene and hepatocellular carcinoma Journal of Biology and Medicine 2003 76 55 62 PMC2582696 15369632

[B22] Nguyen A Jing Z Mahoney PS Davis R Sikka SC Agrawal KC Abdel-Mageed AB In vivo gene expression profile analysis of metallothionein in renal cell carcinoma Cancer Letters 2000 160 133 140 10.1016/S0304-3835(00)00534-6 11053642

[B23] Somji S Sens MA Lamm DL Garrett SH Sens DA Metallothionein isoform 1 and 2 gene expression in the human bladder: evidence for upregulation of MT-1X mRNA in bladder cancer Cancer Detection and Prevention 2001 25 62 75 11270423

[B24] Garrett SH Sens MA Shukla D Flores L Somji S Todd JH Sens DA Metallothionein isoform 1 and 2 gene expression in the human prostate: downregulation of MT-1X in advanced prostate cancer Prostate 2000 43 125 135 10.1002/(SICI)1097-0045(20000501)43:2<125::AID-PROS7>3.0.CO;2-S 10754528

[B25] Li Z Stonehuerner J Devlin RB Huang YC Discrimination of vanadium from zinc using gene profiling in human bronchial epithelial cells Environmental Health Perspectives 2005 113 1747 1754 16330358 10.1289/ehp.7947 PMC1314916

[B26] Yap Y Zhang X Smith D Soong R Hill J Molecular gene expression signature patterns for gastric cancer diagnosis Computational Biology and Chemistry 2007 31 275 287 10.1016/j.compbiolchem.2007.06.001 17631416

[B27] Sorlie T Perou CM Tibshirani R Aas T Geisler S Johnsen H Hastie T Eisen MB Rijn M van de Jeffrey SS Thorsen T Quist H Matese JC Brown PO Botstein D Eystein Lonning P Borresen-Dale AL Gene expression patterns of breast carcinomas distinguish tumor subclasses with clinical implications PNAS 2001 98 10869 10874 10.1073/pnas.191367098 11553815 PMC58566

[B28] Dronkert ML de Wit J Boeve M Vasconcelos ML van Steeg H Tan TLR Hoeijmakers JHJ Kanaar R Disruption of mouse SNM1 causes increased sensitivity to the DNA interstrand cross-linking agent mitomycin C Molecular and Cellular Biology 2000 20 4553 4561 10.1128/MCB.20.13.4553-4561.2000 10848582 PMC85844

[B29] Bae I Fan S Meng Q Rih J Kim H Kang H Xu J Goldberg ID Jaiswal AK Rosen EM BRCA1 induces antioxidant gene expression and resistance to oxidative stress Cancer Research 2004 64 7893 7909 10.1158/0008-5472.CAN-04-1119 15520196

[B30] Swindell WR Gene expression profiling of long-lived dwarf mice: longevity-associated genes and relationships with diet, gender and aging BMC Genomics 2007 8 353 10.1186/1471-2164-8-353 17915019 PMC2094713

[B31] Frias C Garcia-Aranda C De Juan C Moran A Ortega P Gomez A Hernando F Lopez-Asenjo JA Torres AJ Benito M Iniesta P Telomere shortening is associated with poor prognosis and telomerase activity correlates with DNA repair impairment in non-small cell lung cancer Lung Cancer 2008 60 416 425 10.1016/j.lungcan.2007.11.001 18077053

[B32] Efron B Tibshirani RJ An Introduction to the Bootstrap 1994 Chapman & Hall/CRC

[B33] Ma S Huang J Regularized ROC method for disease classification and biomarker selection with microarray data Bioinformatics 2005 21 4356 4362 10.1093/bioinformatics/bti724 16234316

[B34] Ma S Song X Huang J Regularized binormal ROC method in disease classification using microarray data BMC Bioinformatics 2006 7 253 10.1186/1471-2105-7-253 16684357 PMC1513612

[B35] Gui J Li H Threshold gradient descent method for censored data regression with applications in pharmacogenomics Proceedings of Pacific Symposium on Biocomputing 2005 10 272 283 full_text 10.1142/9789812702456_0026 15759633

[B36] Hastie T Tibshirani RJ Friedman J The Elements of Statistical Learning: Data Mining, Inference, and Prediction 2003 Verlag: Springer

[B37] Ma S Huang J Regularized gene selection in cancer microarray meta-analysis BMC Bioinformatics 2009 10 1 10.1186/1471-2105-10-1 19118496 PMC2631520

[B38] Boer JM Huber WK Sultmann H Wilmer F von Heydebrecl A Haas S Korn B Gunawan B Vente A Fuzesi L Vingron M Poustka A Identification and classification of differentially expressed genes in renal cell carcinoma by expression profiling on a global human 31,500-element cDNA array Genome Research 2001 11 1861 1870 11691851 10.1101/gr.184501 PMC311168

[B39] Chen X Cheung ST So S Fan ST Barry C Higgins J Lai KM Ji J Dudoit S Ng IO Rijn M Van De Botstein D Brown PO Gene expression patterns in human liver cancers Molecular Biology of the Cell 2002 13 1929 1939 10.1091/mbc.02-02-0023. 12058060 PMC117615

[B40] Bhattacharjee A Richards WG Staunton J Li C Monti S Vasa P Ladd C Beheshti J Bueno R Gillette M Loda M Weber G Mark EJ Lander ES Wong W Johnson BE Golub TR Sugarbaker DJ Meyerson M Classification of human lung carcinomas by mRNA expression profiling reveals distinct adenocarcinoma subclasses PNAS 2001 98 13790 13795 10.1073/pnas.191502998 11707567 PMC61120

[B41] Iacobuzio-Donahue CA Maitra A Olsen M Lowe AW van Heek NT Rosty C Walter K Sato N Parker A Ashfaq R Jaffee E Ryu B Jones J Eshleman JR Yeo CJ Cameron JL Kern SE Hruban RH Brown PO Goggins M Exploration of global gene expression patterns in pancreatic adenocarcinoma using cDNA microarrays American Journal of Pathology 2003 162 1151 1162 12651607 10.1016/S0002-9440(10)63911-9 PMC1851213

[B42] Singh D Febbo PG Ross K Jackson DG Manola J Ladd C Tamayo P Renshaw AA D'Amico AV Richie JP Lander ES Loda M Kantoff PW Golub TR Sellers WR Gene expression correlates of clinical prostate cancer behavior Cancer Cell 2002 1 203 209 10.1016/S1535-6108(02)00030-2 12086878

[B43] Chen X Leung SY Yuen ST Chu KM Ji J Li R Chan AS Law S Troyanskaya OG Wong J So S Botstein D Brown PO Variation in gene expression patterns in human gastric cancers Molecular Biology of the Cell 2003 14 3208 3215 10.1091/mbc.E02-12-0833 12925757 PMC181561

